# Association between the severity of metabolic dysfunction-associated fatty liver disease and the risk of colorectal neoplasm: a systematic review and meta-analysis

**DOI:** 10.1186/s12944-022-01659-1

**Published:** 2022-06-06

**Authors:** Yunqing Zeng, Ruyue Cao, Ziwen Tao, Yanjing Gao

**Affiliations:** Department of Gastroenterology, Qilu Hospital of Shandong University, 107 West Wen Hua Road, Jinan, 250012 Shandong China

**Keywords:** Metabolic dysfunction-associated fatty liver disease, Colorectal adenoma, Colorectal neoplasm, Severity, Meta-analysis

## Abstract

**Background:**

The severity of metabolic dysfunction-associated fatty liver disease (MAFLD) reportedly plays a part in the etiology of colorectal tumors. However, there is no consensus.

**Methods:**

Studies relevant with the impact of MAFLD severity on the risk of colorectal neoplasms published before 24th April 2022 were screened. The pooled odds ratio (OR) with corresponding 95% confidence intervals (95% CI) was obtained using standard and cumulative meta-analyses. Subgroup, meta-regression, and sensitivity analyses were carried out to identify heterogeneity.

**Results:**

Fourteen studies with data from 37,824 MAFLD patients were included. The prevalence of colorectal neoplasms escalated with the progression of MAFLD compared to simple steatosis (OR = 1.93; 95% CI = 1.42–2.62). The magnitude and direction of the effect on these outcomes remained largely constant over time. Even after limiting the meta-analysis to 8 studies with available adjusted OR (aOR), the findings still suggested that MAFLD severity was positively related to colorectal neoplasms (aOR = 3.03; 95% CI = 2.02–4.53). Severe MAFLD was more likely to cause left colon tumors (OR = 3.86, 95% CI = 2.16–6.91) than right colon neoplasms (OR = 1.94, 95% CI = 1.15–3.28).

**Conclusion:**

The severity of MAFLD was independently related to colorectal neoplasms and severe MAFLD was more likely to cause left colon tumors.

**Supplementary Information:**

The online version contains supplementary material available at 10.1186/s12944-022-01659-1.

## Introduction

Metabolic dysfunction-associated fatty liver disease (MAFLD), previously named non-alcoholic fatty liver disease (NAFLD), involves approximately 25 % of the adults worldwide [1]. MAFLD was significantly associated with a majority of tumorigenic cases (90%), especially colorectal neoplasms which are also common worldwide [2–4]. Therefore, MAFLD causes considerable health and economic burden globally and frequently leads to inferior quality of life. MAFLD includes two histologically different phases with distinct prognoses: non-alcoholic fatty liver (NAFL) and nonalcoholic steatohepatitis (NASH); the latter encompasses different liver tissue lesions, including fibrosis, cirrhosis, and liver cancer [5].

The colorectal area is divided anatomically into the left colon and the right colon, which is separated by the splenic flexure. The definition of advanced colorectal neoplasia is an adenomatous polyp with a diameter of more than 10 mm and/or villous histology and/or high-grade dysplasia or adenocarcinoma.

Many systematic reviews have shown the link between MAFLD and a high risk of colorectal tumors [6–9]. Only two of them briefly assessed the association between the severity of MAFLD and colorectal tumors as a secondary research objective, and only three and seven studies were respectively included in the two meta-analyses [6, 8]. There is still some uncertainty regarding whether the presence of severe steatosis, NASH or advanced fibrosis is more likely to cause colorectal neoplasms compared to mild liver disease [10]. Here, a meta-analysis was conducted for the first time to uncover the potential relationship between different severities of MAFLD, including hepatic steatosis, inflammation and fibrosis, and colorectal neoplasms (colorectal adenomas or/and advanced colorectal neoplasia), which may promote the prevention and detection of colorectal neoplasms. This meta-analysis also evaluated the site-specific effects of the varying severity of MAFLD on colorectal tumors.

## Methods

This meta-analysis was reported following the guidelines of the Meta-analysis Of Observational Studies in Epidemiology [11]. Registration of the study protocol was done in advance (NO. CRD42021269830).

### Methodology of searching

Studies published on PubMed, EMBASE, Cochrane Library, Web of Science and China National Knowledge Infrastructure (CNKI) from inception to 24th April 2022 were retrieved using various combinations of MeSH and non-MeSH terms related to MAFLD and colorectal neoplasm. The search strategy details are shown in Supplemental Table 1. Language and region were not restricted. To search for eligible studies fully, references from relevant articles were also reviewed.

### Study selection

Criteria for eligibility included the following: 1) observational studies (cross-sectional, case-control, or cohort studies) that investigated the association between the severity of MAFLD and colorectal tumors; 2) odds ratio (OR) with 95% confidence interval (CI), or enough raw data to calculate OR with 95% CI were provided; 3) colorectal adenomas and advanced colorectal neoplasia were confirmed by colonoscopy; 4) MAFLD was diagnosed via imaging or biopsy; 5) MAFLD severity was assessed by biopsy, imaging steatosis degree or non-invasive fibrosis scoring systems; 6) no restrictions on race, sex, ethnicity or comorbidities of research subjects; 7) due to the lack of relevant studies, congress abstracts that met the above inclusion criteria were also incorporated; 8) when studies on the same population were published multiple times, only the most recent or comprehensive publication was chosen.

The criteria for excluding studies were as follows: 1) laboratory studies, letters, summaries, reviews, meta-analyses, commentaries, and case reports; 2) studies that include patients with other competing causes (viral infections, drugs, alcohols) of chronic liver diseases; 3) studies where participants were candidate liver transplant recipients with cirrhosis; 4) duplicate studies; 5) studies conducted in pediatric populations.

Two reviewers independently checked each study. Discussions among the two reviewers and the paper’s other author were held to resolve disagreements.

### Data extraction

Based on a standardized form, the following data were summarized: the number of patients with MAFLD; first author; publication date; sex-related data; country of study; study design; methods used for MAFLD diagnosis; assessment methods for the severity of MAFLD; the outcome of interest (colorectal adenomas or advanced colorectal neoplasia); covariates; Newcastle–Ottawa Scale (NOS)/Agency for Healthcare Research and Quality (AHRQ) scores.

### Quality assessment

Two authors evaluated the quality of the eligible researches separately. Any disagreements were resolved via a re-valuation of the studies by another reviewer. Case-control and cohort studies were appropriate for the NOS scale, while cross-sectional studies were assessed using the AHRQ scale [12]. The NOS evaluates the quality of a study based on 3 criteria: selection, comparability, and outcome. Studies that received a six-star rating or higher were denoted as high quality in this paper. The AHRQ scale grades the quality of articles as “low” (score of 0–3), “moderate” (4–7), or “high” (8–11) based on 11 items [13].

### Statistical analysis

Analysis of the data was performed with Stata version 16.0 SE (Stata Corp, College Station, TX) and Review Manager version 5.3 (RevMan, the Cochrane Collaboration, Oxford, UK). The OR was used as the effect size for binary variables, and each effect size provided its 95% CI. If a study had multiple adjustment models, the one that maximally adjusted the confounding factors were selected. The pooled ORs and the 95% CIs were calculated to show the effect of MAFLD severity on the occurrence of colorectal neoplasm. The final outcomes were visualized as forest plots. Statistical significance was denoted by *P* values below 0.05 (two-sided).

Quantitative heterogeneity was evaluated by Q-based *I*^*2*^, where the Q-statistic was made up of the weighted sum of the squared values of the study effect size deviation from the overall mean effect size. The *I*^*2*^ index measured the proportion of heterogeneity that is unknown or unexplained [14], and *I*^*2*^ > 50% or *P* < 0.05 meant the presence of significant heterogeneity. In the absence of non-negligible heterogeneity, the fixed-effects model was applied to pool studies; otherwise, the random-effects model was selected [15]. Cumulative meta-analysis treated the data as a continuous unity and conducted separate meta-analyses each time a new study was included. It reflected the trend of the estimator of effect size over time to measure the time taken for the research subjects to reach sufficient stability [16]. Subgroup analyses were conducted in order to explain some possible causes of heterogeneity, allowing effect sizes of studies within a subgroup to be compared and assessing if heterogeneity was reduced through subgroup analyses [14]. Meta-regression analysis was conducted to evaluate potential regulatory influences of the variables on between-study heterogeneity [17]. To find the outlier studies and determine the firmness of the original results, sensitivity analyses were carried out based on the removal of one study at a time. The funnel plot, Begg’s test, and Egger’s test were performed to judge the possibility of publication bias [18, 19].

## Results

### Features of selected studies

**Fig. 1:**
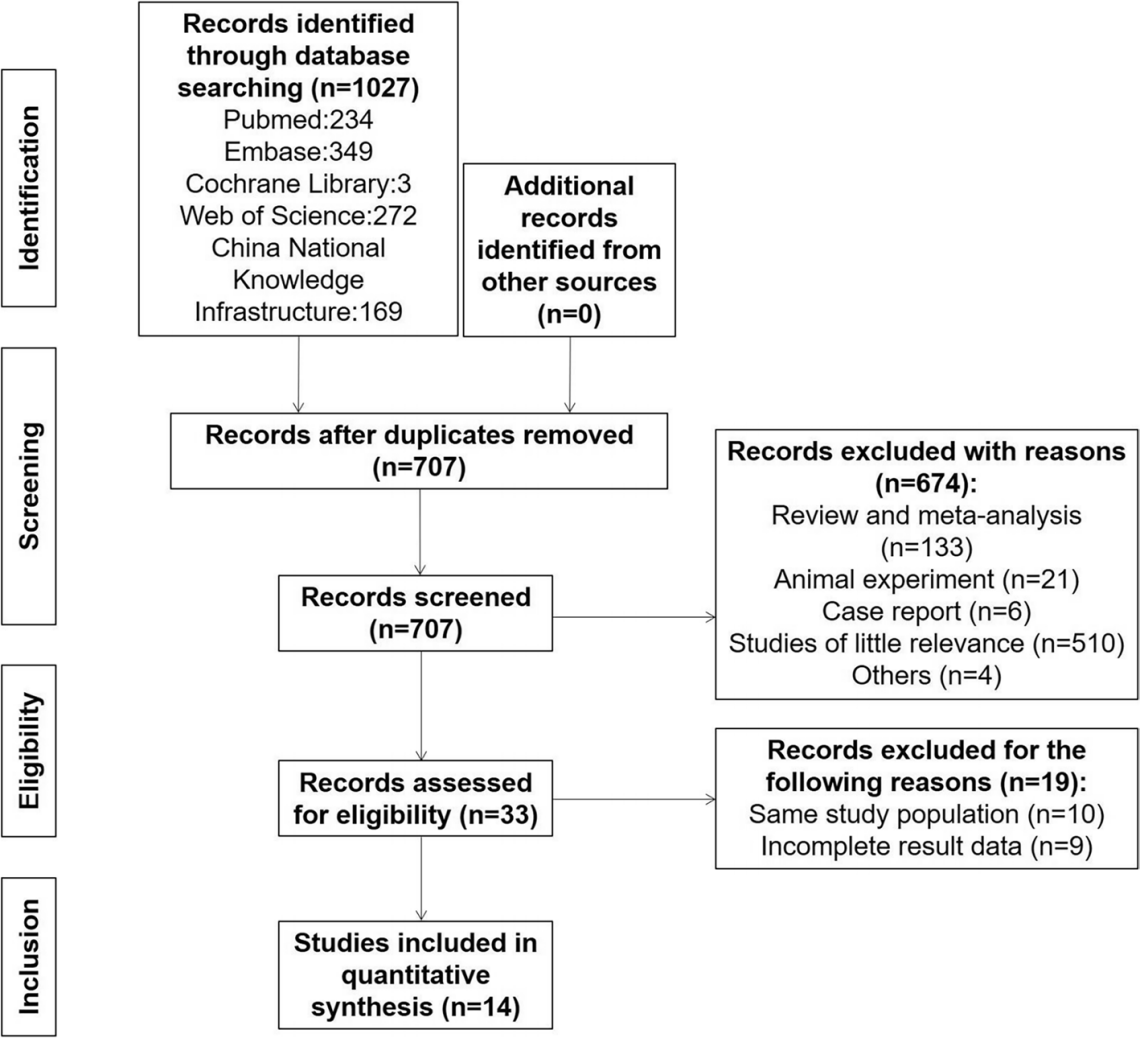
The PRISMA flow diagram

The detailed selection process are presented in Fig. 1. 1027 records in all were retrieved after the initial search (234 from PubMed, 349 from Embase, 3 from the Cochrane library, 272 from the Web of Science, and 169 from CNKI), 320 were duplicate. 674 records were excluded following a careful review of titles and abstracts. Of the remaining 33 articles, 19 met the exclusion criteria. Finally, 14 studies were included.

Table 1 lists the detailed information of the 14 studies [20–32]. Total 37,824 MAFLD patients in these studies all underwent a screening colonoscopy. MAFLD was diagnosed by either biopsy or imaging techniques [liver biopsy, *n* = 4 studies; ultrasonography, *n* = 7 studies; Fibro Touch, *n* = 1 study; Transient Elastography (TE) with Controlled Attenuation Parameter (CAP), *n* = 1 study; biopsy or ultrasonography, *n* = 1 study]. 11 studies were from Asia (South Korea, China, and Thailand), two from North America (the United States), and one from Europe (Romania). Eight studies adjusted potential confounding factors, whereas six studies did not provide the adjusted OR. In order to assess the severity of MAFLD, five studies explored the stage of liver fibrosis, four evaluated the fatty liver grade, and five determined the presence and absence of NASH. Ten cross-sectional studies scored at least eight stars on the AHRQ, one case-control study scored seven stars and three cohort studies scored at least six stars on the NOS.

**Table 1:** The characteristics of the included studies (Page 9; line 139)

Author, year	Study design, country, number of patients with MAFLD	Diagnosis of MAFLD, assessment methods for the severity of MAFLD	Sex-male%, the prevalence of colorectal neoplasms by sex	Main findings	Covariate adjustment(s)	NOS/AHRQ
Liu, 2022 [20]	Cross-sectional study, China, 331	Ultrasonography, non-invasive fibrosis score	N/A	The degree of fibrosis in MAFLD is closely related to the prevalence of colorectal adenomatous polyp and high-risk adenoma.	Age, sex, and BMI	9
Seo, 2021 [31]	Cross-sectional study, Korea, 1127	Ultrasonography, non-invasive fibrosis score	79.6, 33.7%-male; 31.3%-female	MAFLD with advanced fibrosis was associated with an increased risk of colorectal adenoma.	Sex, smoking, and visceral fat area	9
Chuan, 2020 [21]	Cross-sectional study, China, 78	Fibro Touch, Fibro Touch	67.9% (severe MAFLD group 73.1%, mild or moderate MAFLD group 65.4%), N/A	The prevalence of adenomas was similar when comparing patients with CAP≥295 dB/m vs. 240 dB/m ≤ CAP< 295 dB/m.	N/A	8
Blackett, 2020 [20]	Cross-sectional study, the United States, 123	Biopsy, biopsy	49.6, 50.8%-male; 30.7%-female	The prevalence of adenomas was similar when comparing patients with no NASH versus NASH.	Age, sex, endoscopist, hyperlipidemia, diabetes, obesity, and colonoscopy indication	9
Cho, 2019 [22]	Cohort study, Korea, 379	Biopsy, biopsy	N/A	The prevalence of adenomas and advanced neoplasia was similar when comparing patients with NAFL versus NASH.	N/A	6
Kim, 2019 [23]	Cross-sectional study, Korea, 2395	Ultrasonography, non-invasive fibrosis score	71.3%	MAFLD patients with advanced fibrosis had a significantly higher risk for colorectal adenomas than those without advanced fibrosis.	Age, sex, obesity, smoking, hypertension, DM, hyperlipidemia, and metabolic syndrome	9
Kim, 2018 [24]	Cohort study, Korea, 8721	Ultrasonography, non-invasive fibrosis score	71.1%, 85.7 per 100,000 person-years -male; 30.3 per 100,000 person-years -female	The severity of MAFLD was not related to colorectal cancer	Age, sex, smoking status, diabetes, hypertension, GGT, HDL cholesterol, LDL cholesterol, and triglycerides	8
Ahn, 2017 [32]	Cross-sectional study, Korea, 9501	Ultrasonography, non-invasive fibrosis score	N/A	When compared to MAFLD patients with mild liver disease, the ORs for advanced colorectal neoplasia were higher for those with advanced fibrosis.	Age, sex, BMI, smoking, alcohol, aspirin use, fasting plasma glucose, first-degree family history of colorectal cancer, serum lipids, systolic blood pressure, drugs	9
Piyachaturawat, 2016 [25]	Cross-sectional study, Thailand, 161	TE-CAP, TE-CAP	N/A	The prevalence of adenomas and advanced adenomas was similar when comparing patients with fatty liver grade Severe vs. Mild to moderate.	N/A	7
Lee, 2016 [26]	Cross-sectional study, Korea, 14,655	Ultrasonography, ultrasonography	86.3% (severe MAFLD group 93.9%, mild or moderate MAFLD group 86.3%), N/A	The prevalence of adenomas and advanced neoplasia was similar when comparing patients with fatty liver grade Severe vs. Mild to moderate.	N/A	8
Yang, 2014 [27]	Cross-sectional study, China, 74	Ultrasonography, ultrasonography	90.5% (severe or moderate MAFLD group 90.6%, mild MAFLD group 90.5%), N/A	The prevalence of adenomas was similar when comparing patients with fatty liver grade Moderate to severe vs. Mild	N/A	8
Tantau, 2014 [28]	Case-control study, Romania, 50	Liver biopsy or abdominal ultrasounds, liver biopsy or abdominal ultrasounds	N/A	NASH is independently related to the prevalence of colorectal adenomas.	Demographic and metabolic factors	7
Wong, 2011 [29]	Cross-sectional study, China, 135	Biopsy, biopsy	54.8%, N/A	NASH is independently related to the prevalence of colorectal adenomas and advanced neoplasia.	Demographic and metabolic factors	9
Touzin, 2011 [33]	Cohort study, the United States, 94	Biopsy, biopsy	62.8% (NASH 65.5%, non-NASH 61.5%), N/A	The prevalence of adenomas was similar when comparing patients with NASH vs. Non-NASH.	N/A	6

### Main outcomes of standard and cumulative meta-analysis

**Fig. 2:**
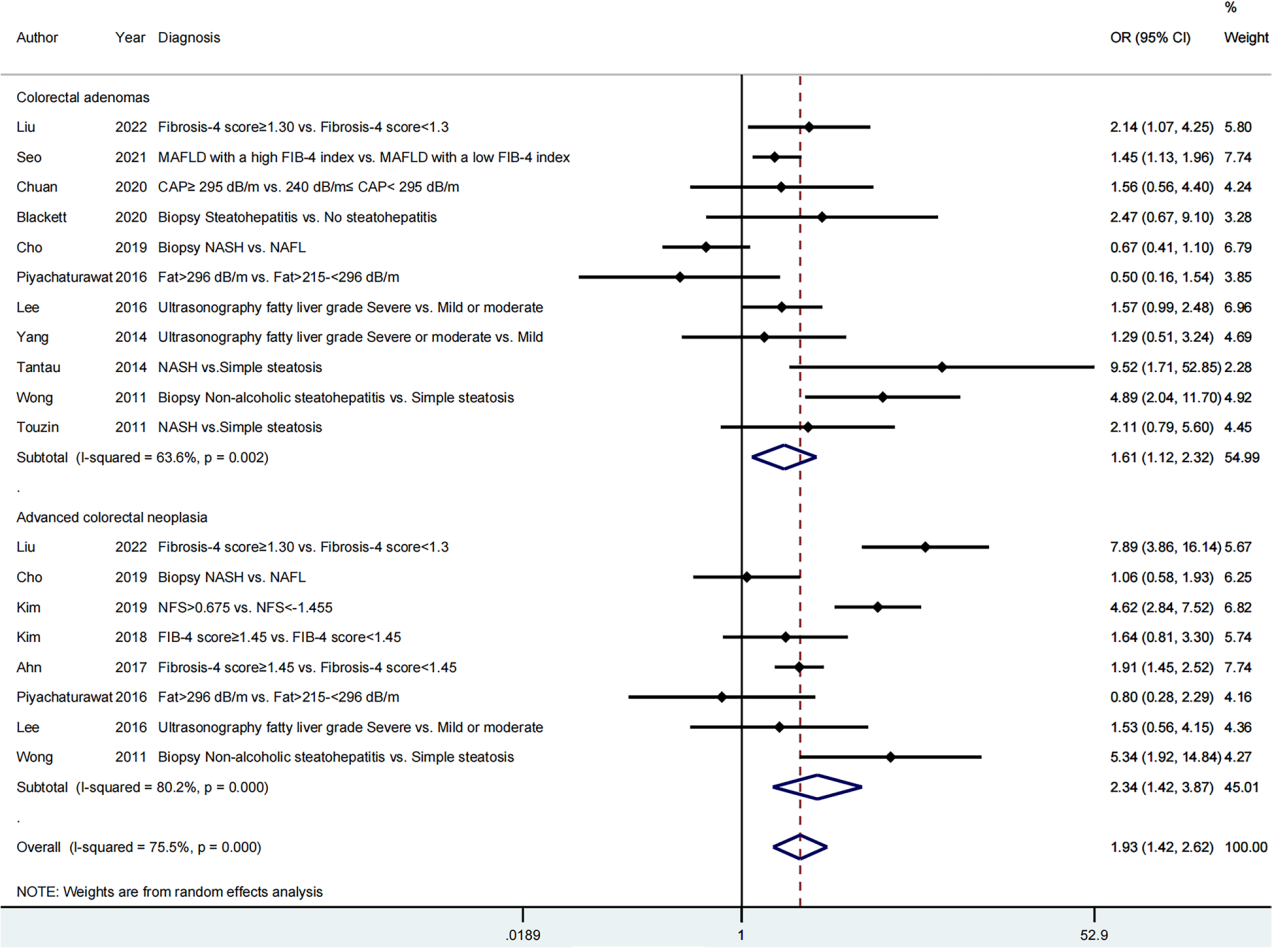
Forest plot of the relationship between the severity of MAFLD and colorectal neoplasms

**Fig. 3:**
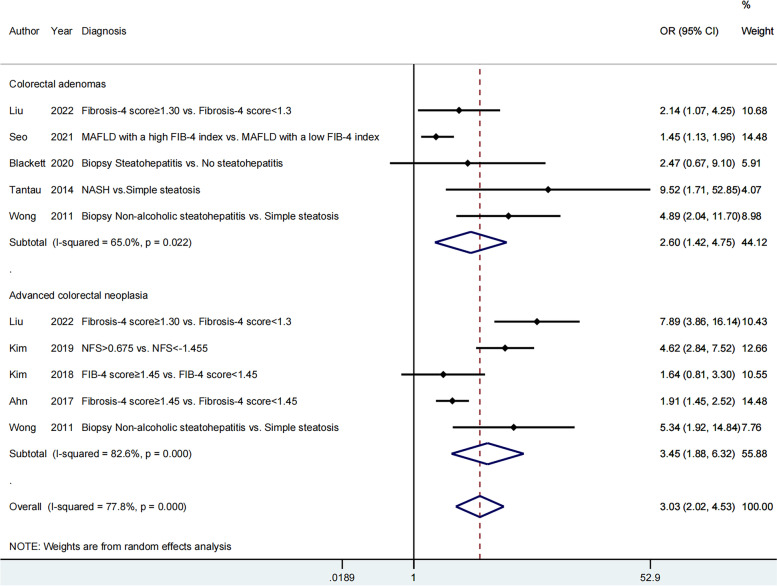
The meta-analysis of eight studies providing the aOR

**Fig. 4:**
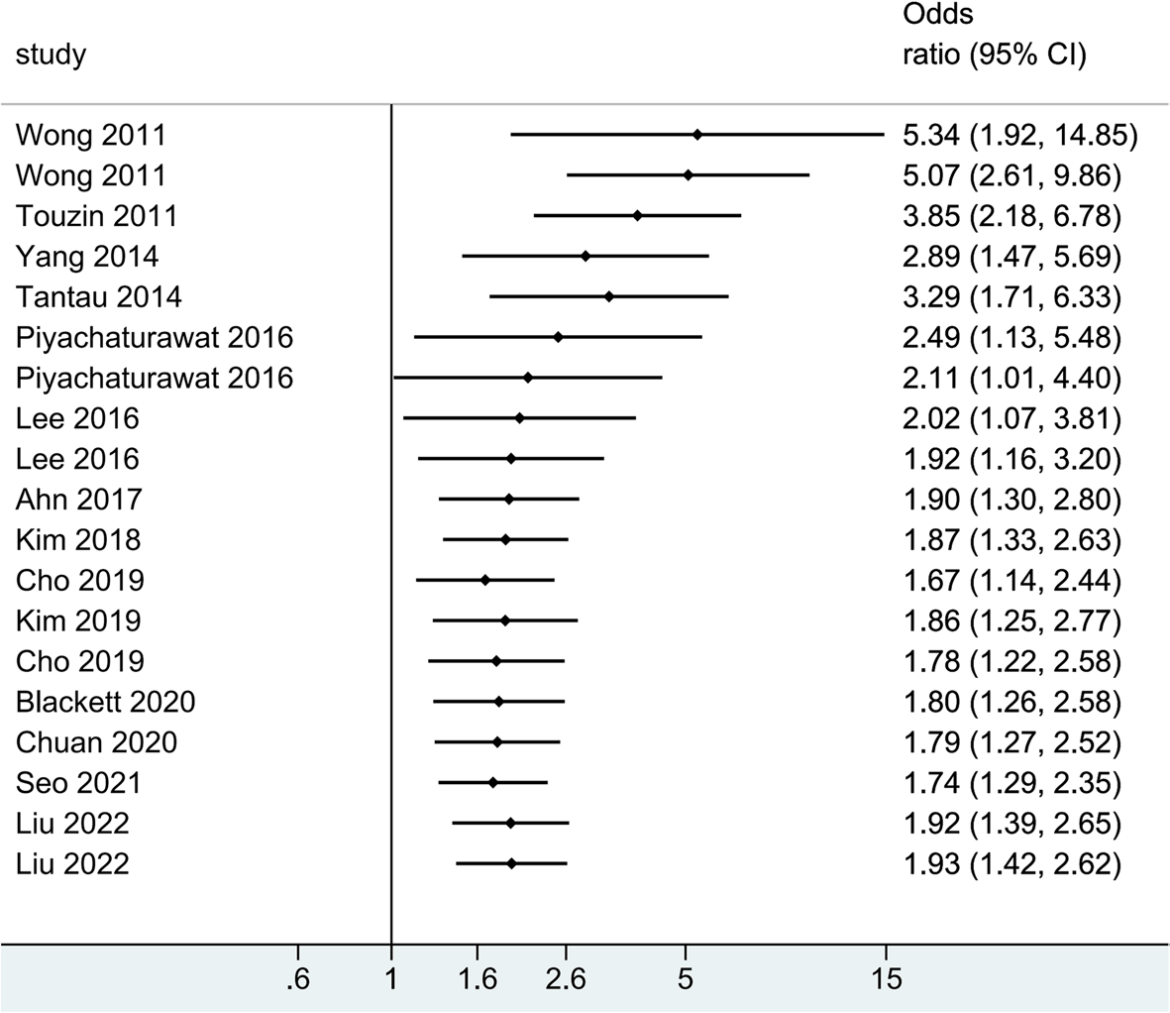
Cumulative meta-analysis of 14 studies

Fourteen articles were included to assess the impact of severity of hepatic steatosis and fibrosis on the occurrence of colorectal neoplasms; 11 articles [20–22, 25–31] on colorectal adenomas and eight studies [22–26, 29, 30, 32] on advanced colorectal neoplasia. The pooled effect estimate was statistically significant (OR = 1.93; 95% CI = 1.42–2.62), along with obvious heterogeneity (*I*^*2*^ = 75.5 > 50%, *P* = 0.000 < 0.05; Fig. 2). Hence, the random-effects model was selected throughout this study. Additionally, the pooled effect estimate showed a higher risk of both colorectal adenomas (OR = 1.61; 95% CI = 1.12–2.32) and advanced colorectal neoplasia (OR = 2.34; 95% CI = 1.42–3.87) in patients with greater severity of MAFLD. The meta-analysis of eight studies, which provided the aOR, also revealed that severe MAFLD had a positive impact on colorectal adenomas (aOR = 2.60; 95% CI = 1.42–4.75) as well as advanced colorectal neoplasia (aOR = 3.45; 95% CI = 1.88–6.32). However, the heterogeneity was still high (Fig. 3). A cumulative meta-analysis showed that this evidence had been available since 2011 and that additional data had provided further accuracy of point estimates, without changing either the direction or magnitude of the effect (Fig. 4).

### Subgroup analyses

Study design, study region, and the classification methods for the severity of MAFLD in the included studies differed greatly, all of which could be underlying factors affecting study outcomes. Therefore, the subgroups based on the above factors were established to determine the source of heterogeneity.

#### Study design

**Fig. 5:**
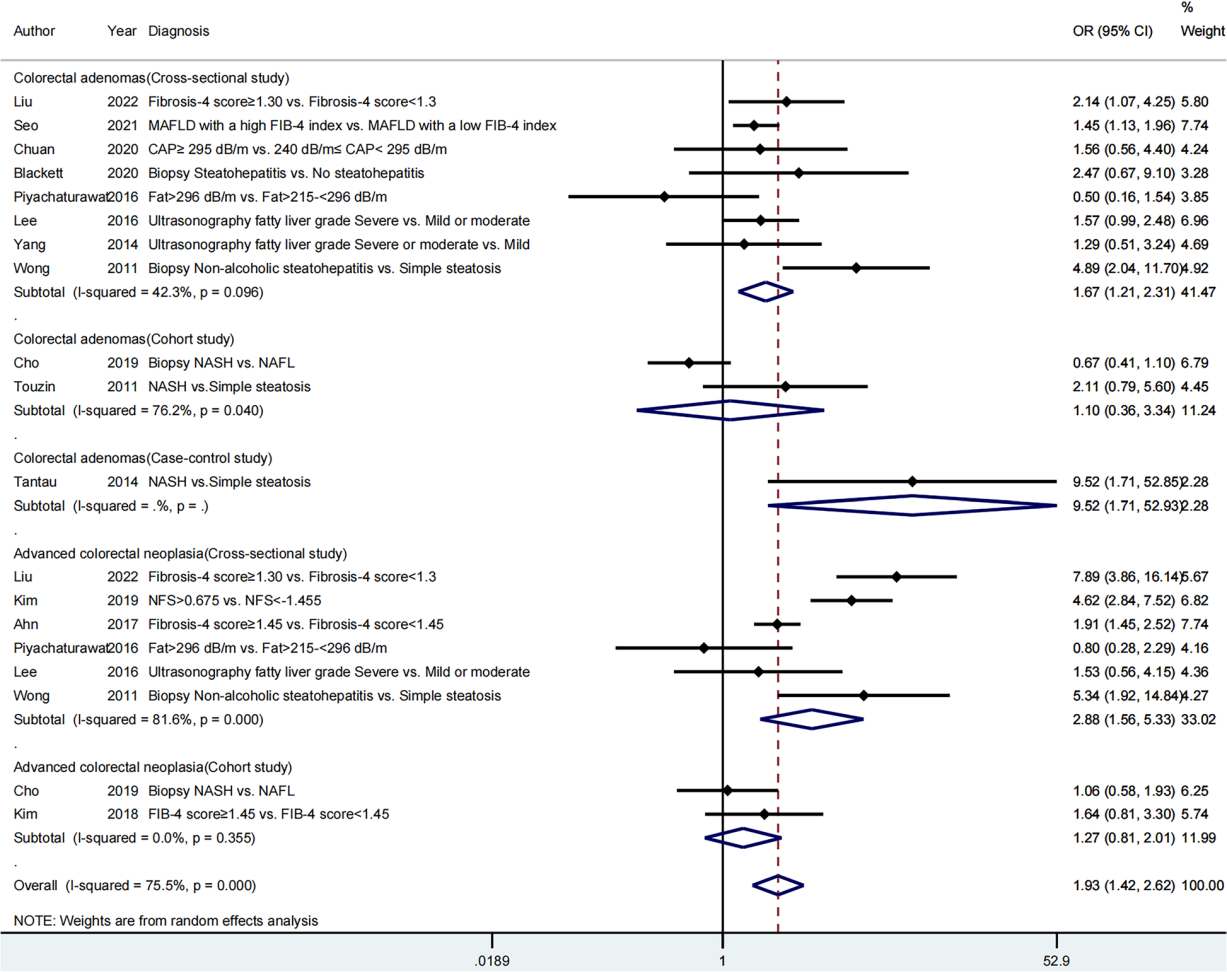
Subgroup analysis by study design

Higher prevalence of colorectal adenomas (OR = 1.67, 95% CI = 1.21–2.31, *I*^*2*^ = 42.3%, *P* = 0.096) and advanced colorectal neoplasia (OR = 2.88, 95% CI = 1.56–5.33, *I*^*2*^ = 81.6%, *P* = 0.000) were found in patients with greater severity of MAFLD than in controls in the cross-sectional studies, whereas no significant differences in the cohort studies were observed. One case-control study relevant to the relationship between severe MAFLD and colorectal adenomas indicated a positive result (OR = 9.52, 95% CI = 1.71–52.93; Fig. 5).

#### Study region

**Fig. 6:**
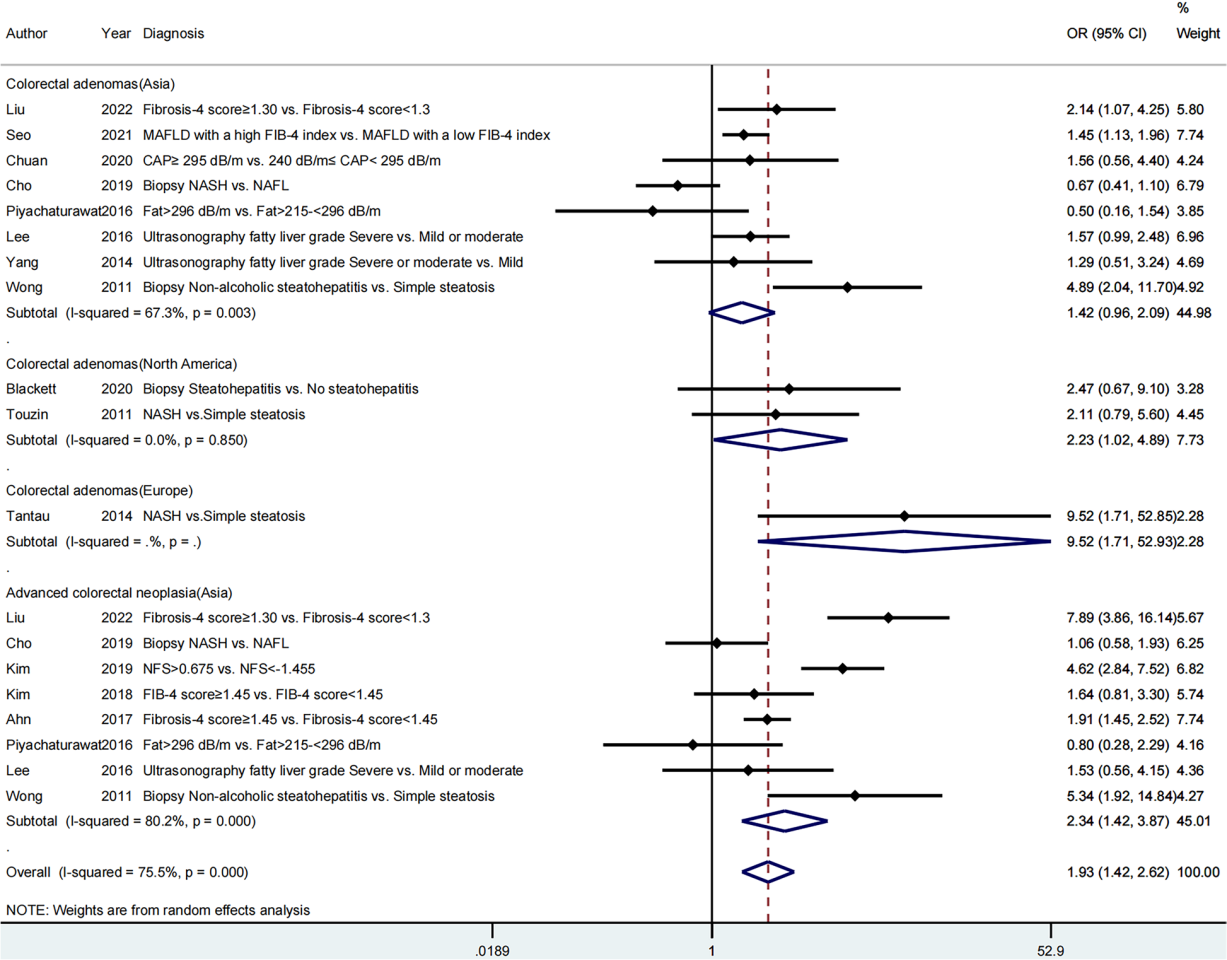
Subgroup analysis by study region

The subgroups of Asia, Europe, and North America were analyzed in accordance with the study region. Severe MAFLD led to an higher prevalence of colorectal adenomas in the subgroups of Europe (OR = 9.52, 95% CI = 1.71–52.93) and North America (OR = 2.23, 95% CI = 1.02–4.89, *I*^*2*^ = 0.0%, *P* = 0.850), but not in the Asia subgroup (OR = 1.42, 95% CI = 0.96–2.09, *I*^*2*^ = 67.3%, *P* = 0.003). However, severe MAFLD seemed more likely to develop advanced colorectal neoplasia in Asian countries with an overall OR of 2.34 (1.42, 3.87) (Fig. 6).

#### Classification methods for the severity of MAFLD

**Fig. 7:**
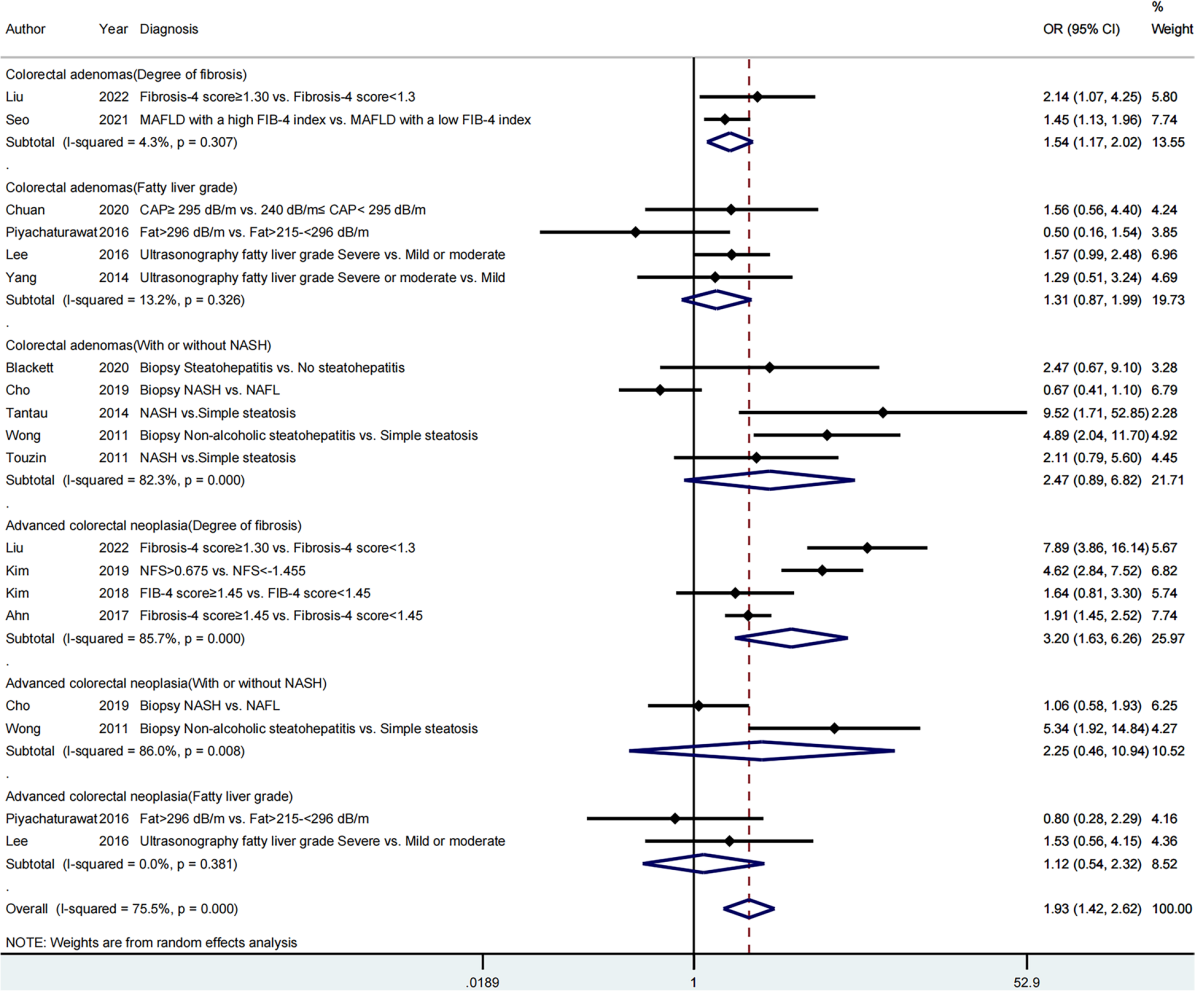
Subgroup analysis by classification methods for the severity of MAFLD

When assessing the severity of MAFLD by the degree of liver fibrosis, the total ORs of colorectal adenomas and advanced colorectal neoplasia were 1.54 [95% CI (1.17–2.02)] and 3.20 [95% CI (1.63–6.26)], respectively. However, when evaluating steatosis grade and the presence and absence of NASH, no significant differences were found for both colorectal adenomas and advanced colorectal neoplasia (Fig. 7).

### Meta-regression

**Fig. 8:**
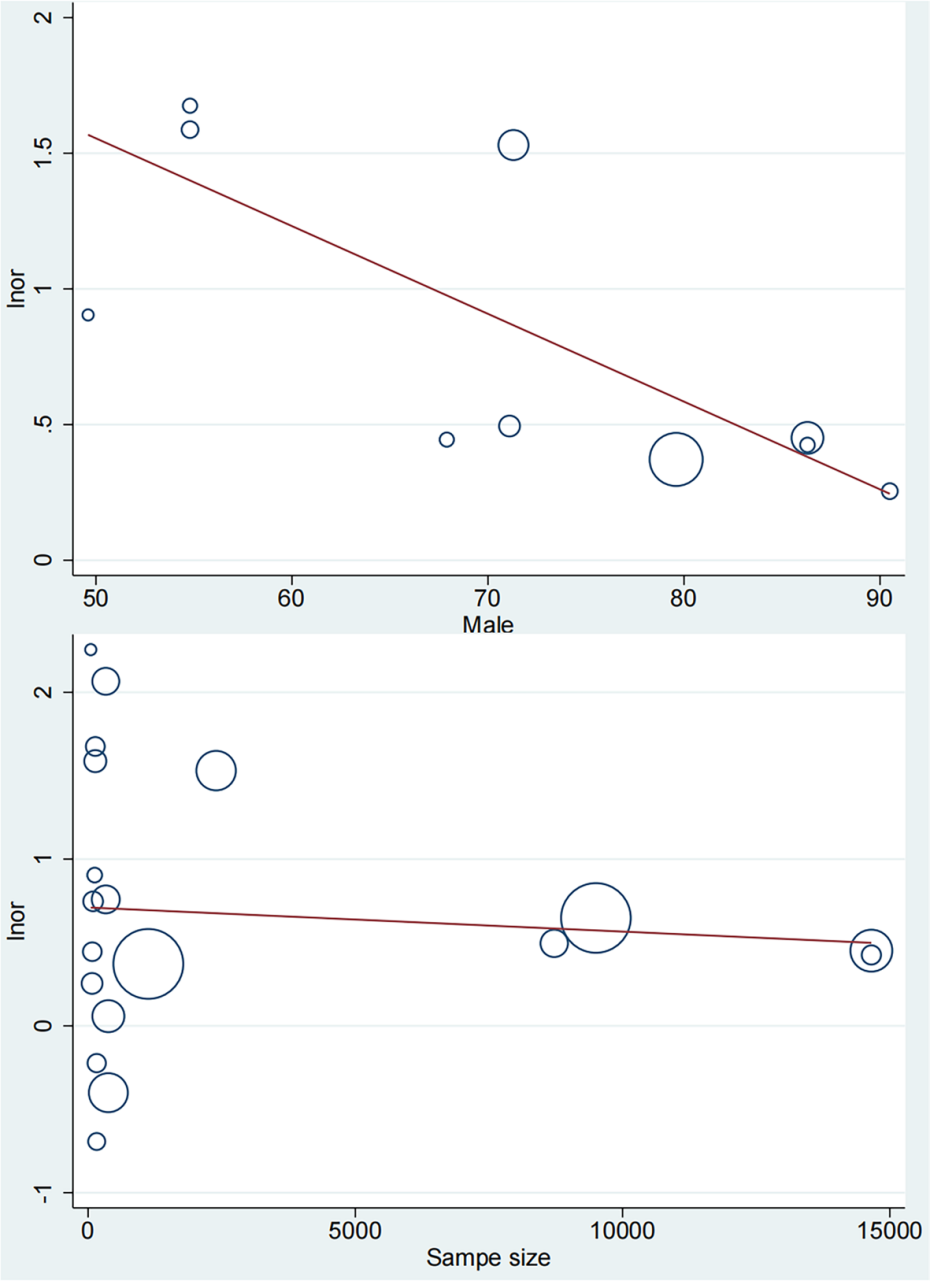
Univariate meta-regression according to sex ratio (**A**); and sample size (**B**)

Since no specific source of heterogeneity could be identified in subgroup analyses, all patients with MAFLD were subjected to univariate meta-regression based on sample size and gender ratio. The findings indicated that the sex ratio played a role in the data heterogeneity (Adjusted *R*^*2*^ = 60.72%; *I*^*2*^ = 38.96%; *P* = 0.030; 95%CI = 0.941–0.996; Fig. 8A). The sample size did not work in the heterogeneity exploration (Fig. 8B). Owing to the lack of relevant reports, meta-regression analyses according to mean age, race, mean transaminase levels, etc., were not conducted.

### Sensitivity analyses

**Fig. 9:**
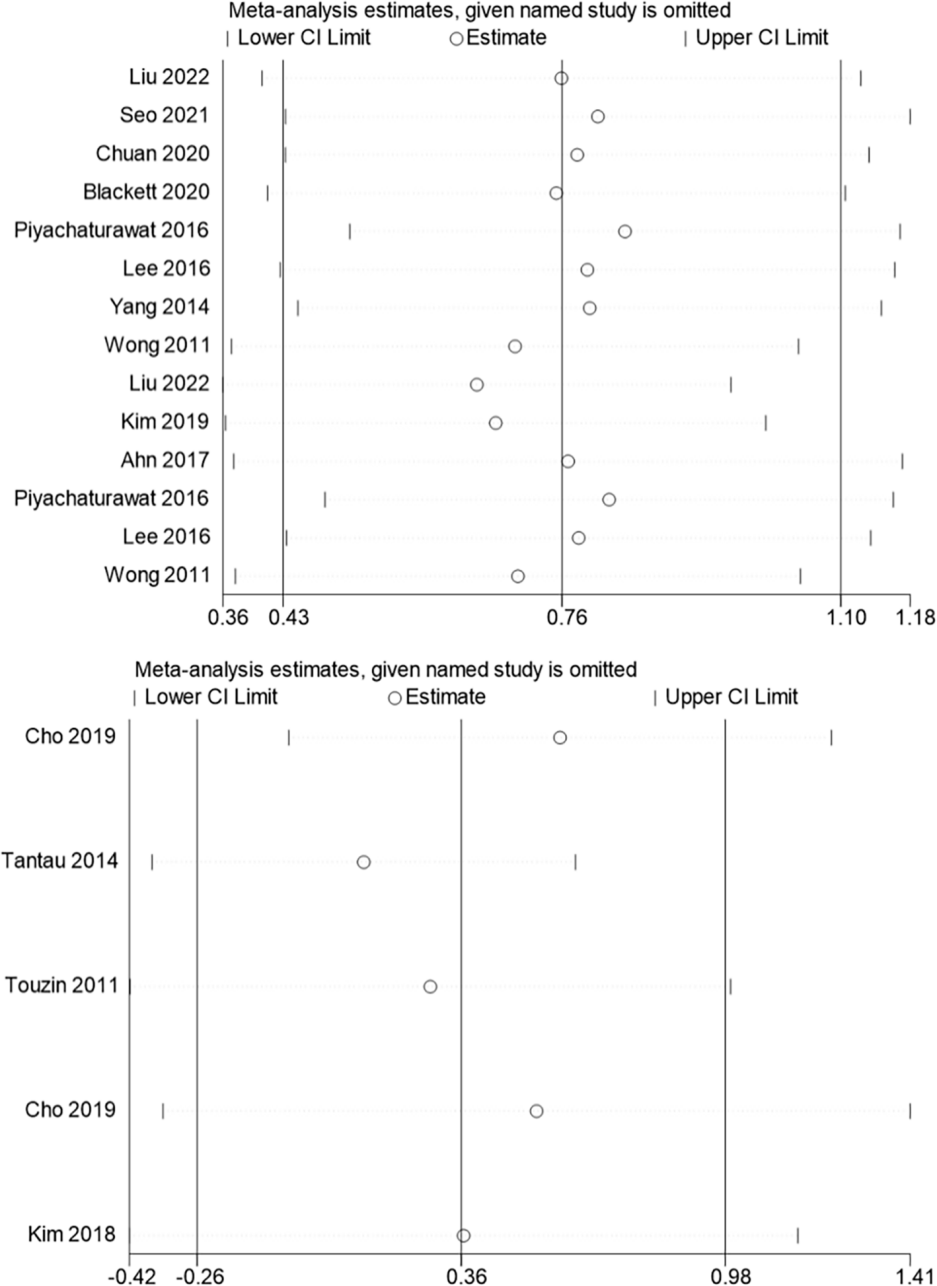
Sensitivity analyses conducted in cross-sectional studies (**A**); and case-control and cohort studies (**B**)

By sequentially eliminating each study, sensitivity analyses were carried out to assess their impact on the overall result. Figure 9 showed that the pooled effect and 95% CI did not change significantly, which indicated the stability of the original results.

### Site-specific prevalence of colorectal tumors

**Fig. 10:**
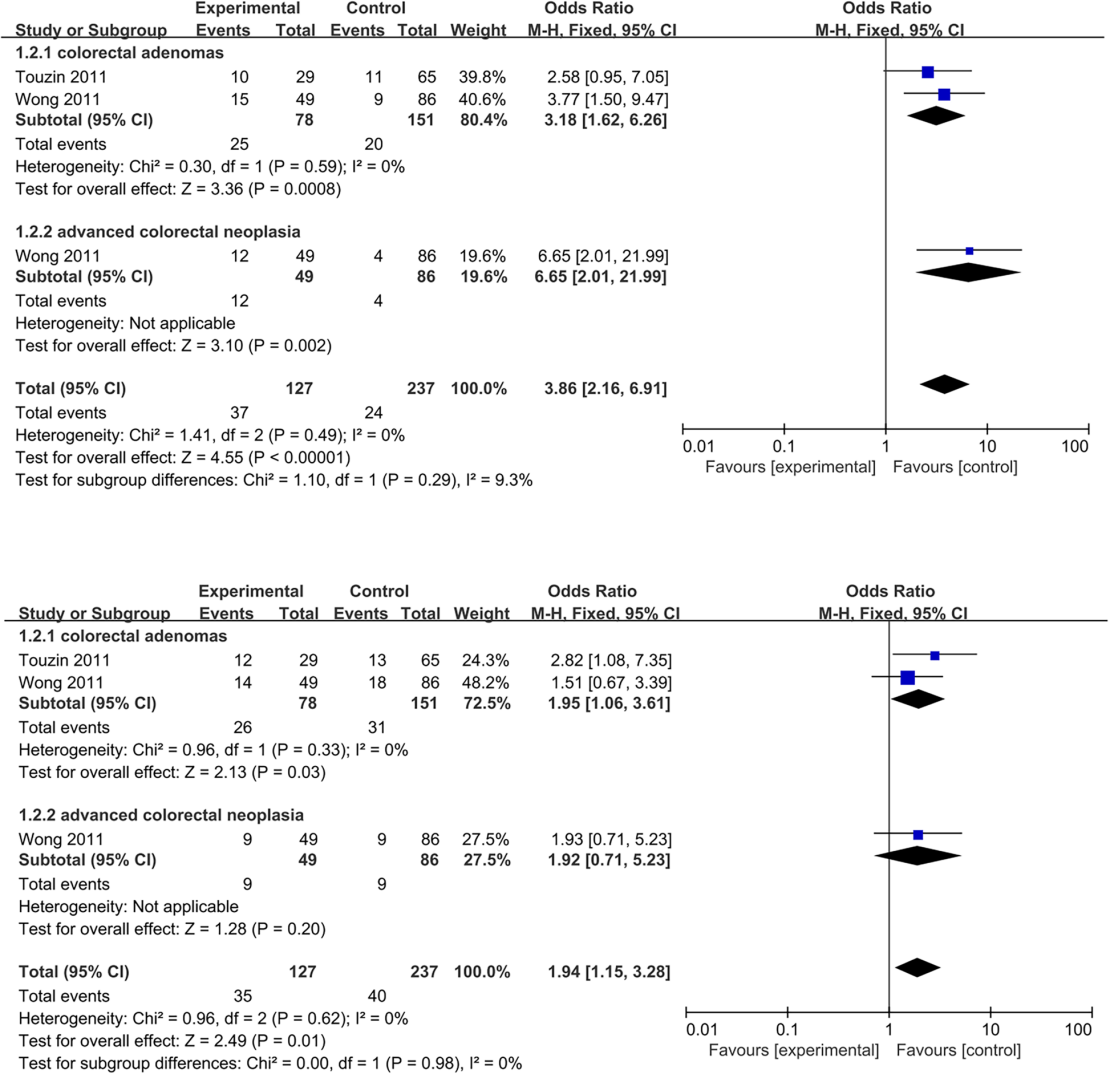
Forest plots of the relationship between the severity of MAFLD and left colon tumors (**A**); and right colon tumors (**B**)

Two studies explored the link between the severity of MAFLD and the location of colorectal adenomas [29, 33]. One study quantified the relationship between MAFLD and the location of advanced colorectal neoplasia [29]. The results revealed that regardless of whether it was on the left or right side, the risk of colorectal tumors in patients with severe liver disease was higher than in controls (Fig. 10A; Fig. 10B). Moreover, left colon tumors were more likely to be caused by severe MAFLD (Left: OR = 3.86, 95% CI = 2.16–6.91, *I*^*2*^ = 0%, *P* = 0.49; Right: OR = 1.94, 95% CI = 1.15–3.28, *I*^*2*^ = 0%, *P* = 0.62).

### Publication bias

**Fig. 11:**
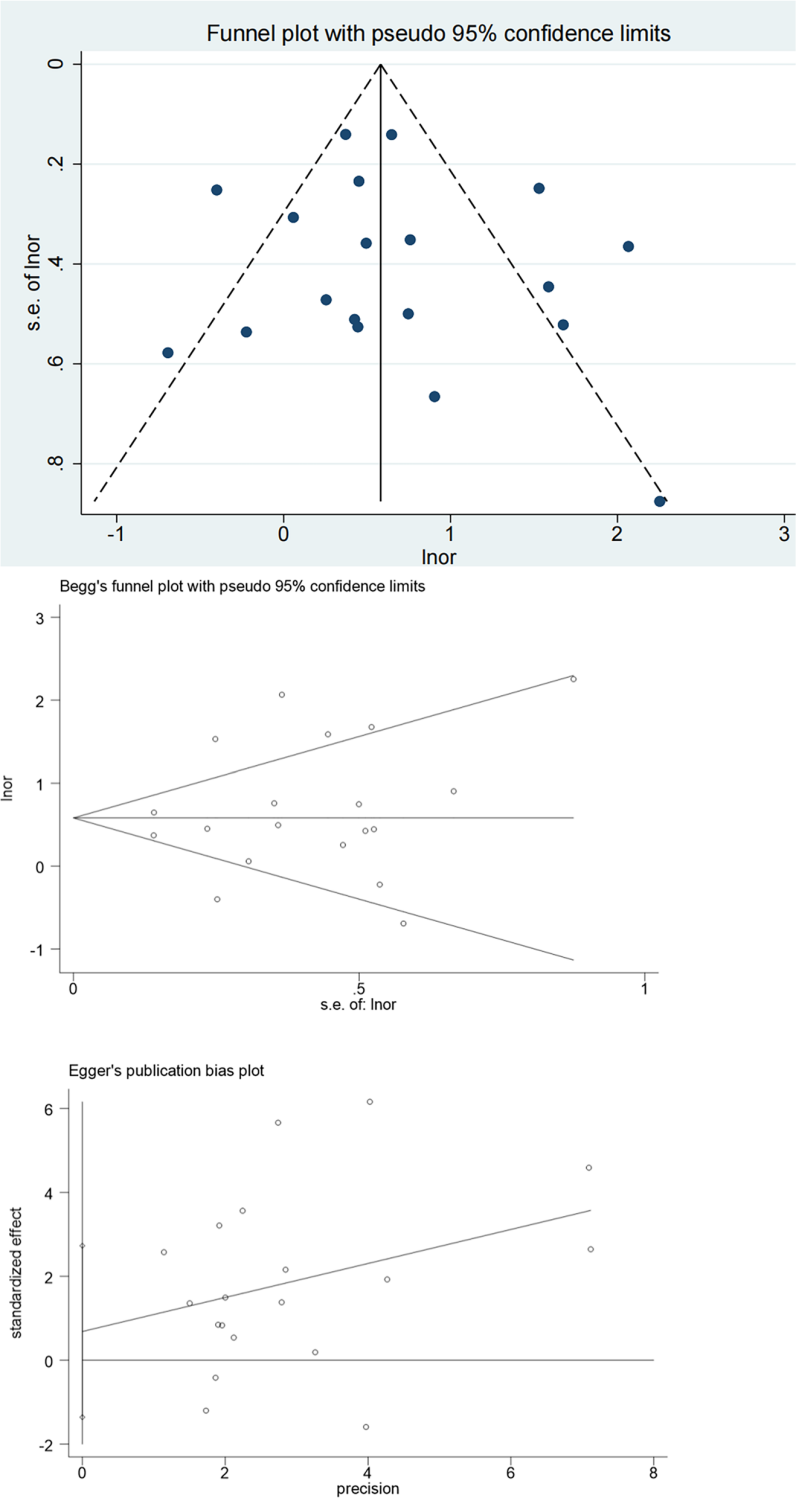
Publication bias of the 14 studies

**Fig. 12:**
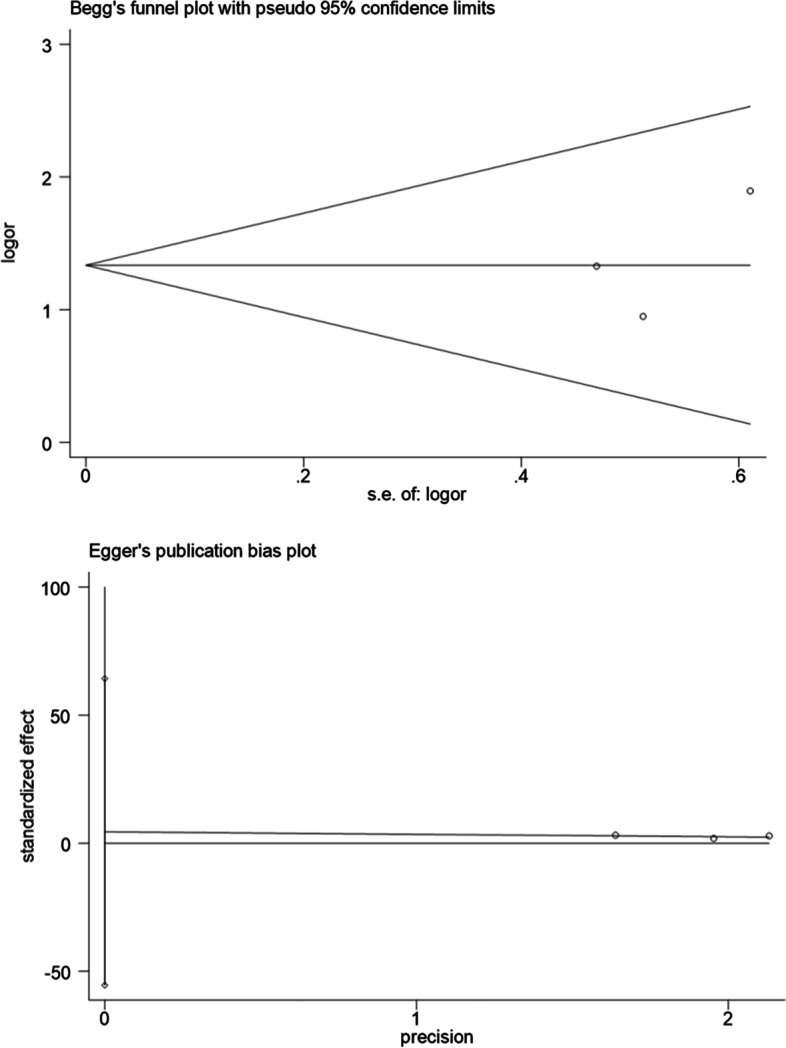
Publication bias of studies exploring the link between the severity of MAFLD and left colon tumors

**Fig. 13:**
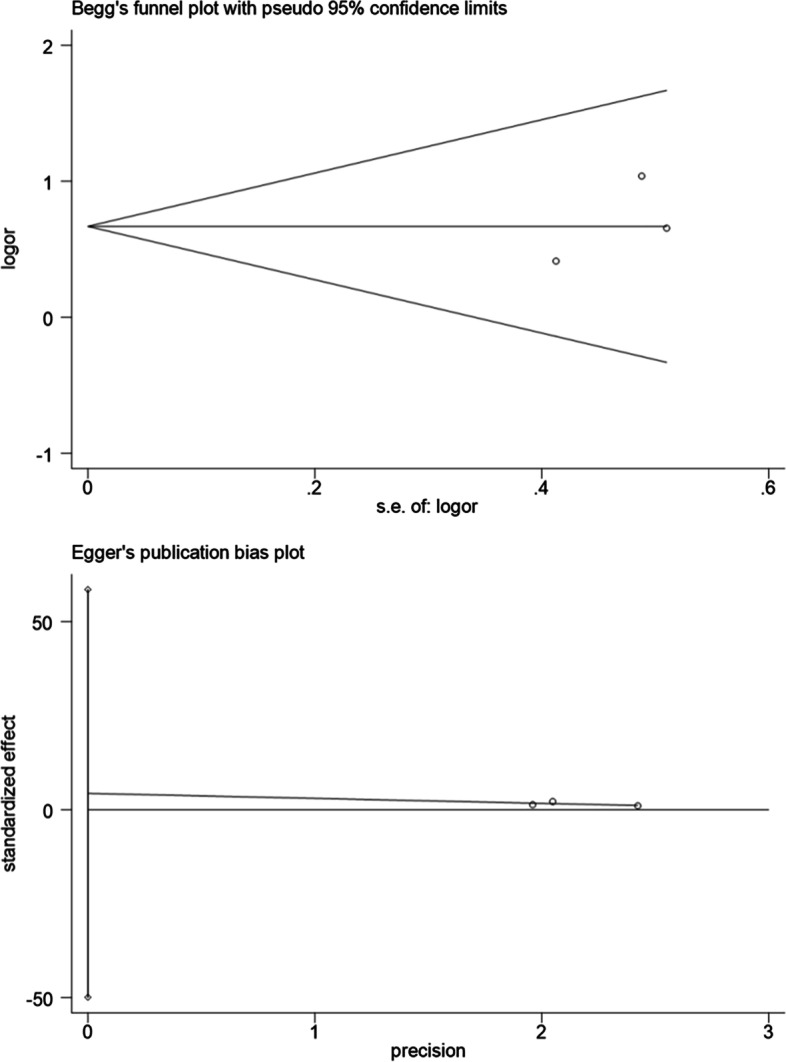
Publication bias of studies exploring the link between the severity of MAFLD and right colon tumors

The funnel plot of pooled OR for colorectal neoplasms showed symmetry (Fig. 11). Begg’s (*P* = 0.889) and Egger’s test (*P* = 0.489) also showed a non-significant results (Fig. 11). As a result of insufficient studies included, the funnel plots were inapplicable. Hence, statistical tests were conducted on the publication bias of pooled OR for tumor location and showed no indications of publication bias (Begg’s test & Left: *P* = 1.000; Begg’s test & Right: *P* = 1.000; Egger’s test & Left: *P* = 0.521; Egger’s test & Right: *P* = 0.497; Fig. 12; Fig. 13).

## Discussion

The clinical and economic burden of MAFLD and colorectal neoplasms is considerable since the prevalence of the two diseases is high among the general public. However, most studies focus on the relationship between MAFLD and colorectal neoplasms. Further researches on the relationship between the severity of MAFLD and colorectal tumors are limited. It is the first research that systematically investigate the prevalence of colorectal neoplasms in patients with different MAFLD severities. Results showed that in comparison to patients with simple steatosis, milder liver fibrosis, and less liver fat, the incidence of colorectal adenomas increased by 1.61 times in severe MAFLD patients, and the incidence of advanced colorectal neoplasia increased by 2.34-fold. These outcomes largely exhibited the same direction and magnitude of effect over time. Furthermore, the pooled effect estimate for eligible studies that were fully adjusted for confounding factors was higher, indicating an independent relationship between the severity of MAFLD and colorectal neoplasms. Additionally, this meta-analysis discovered that left colon tumors are more likely to be caused by severe MAFLD. However, due to the scarcity of related studies, this conclusion was deemed untrustworthy. Additional verification is required.

Numerous studies have demonstrated that four main mechanisms, namely insulin resistance, chronic inflammation, adipocytokines, and intestinal microecology alteration, mediate the association between MAFLD and colorectal adenomas or colorectal cancer (CRC) [34–40]. Hyperinsulinemia due to insulin resistance can both directly stimulate neoplastic growth of the colonic mucosa and indirectly lead to colorectal tumors by increasing insulin-like growth factor-1 level [41, 42]. Other pro-inflammatory cytokines can contribute to the development of MAFLD and colorectal tumors by inducing metabolic liver inflammation and insulin resistance through various complex inflammatory signaling pathways, such as IL-6 and TNFα [43–45]. As adipocytokines, adiponectin and leptin play opposite roles in the proliferation and migration of colorectal tumor cells [41, 43, 46]. When serum adiponectin levels decreases in MAFLD, leptin is more potent to exert a carcinogenic effect [42, 47]. Further, low levels of plasma adiponectin are especially in relation to the risk of KRAS-mutant CRC [48]. Gut microbiota dysbiosis increases intestinal permeability thus causing liver inflammation and damage, accelerates a chronic systemic inflammatory state, as well as produces genotoxins that interfere with the regulation of the intestinal cell cycle [49–51]. The severity of MAFLD is in close relation to the risk of colorectal tumors, possibly because inflammatory state, insulin resistance, decreased serum adiponectin levels, and intestinal bacterial overgrowth are more common and severe with the progression of MAFLD histology [37, 38, 52, 53].

There was significant heterogeneity among the studies included. Subgroup analysis revealed that despite the lack of uniform non-invasive methods for stratifying fibrosis, there was a higher incidence of colorectal neoplasms in MAFLD patients with advanced liver fibrosis than those without. This may be because significant fibrosis implies the end stage of MAFLD. Besides, patients with NASH had a higher risk of colorectal adenomas and advanced colorectal neoplasia than patients with simple steatosis. But this result lack statistical significance, perhaps because of the high heterogeneity among related studies. However, an interesting finding was that severe MAFLD confirmed by imaging techniques did not show any relationship with colorectal adenomas and advanced colorectal neoplasia, and there was almost no heterogeneity among related studies. This might be due to the unreliable classification of the degree of liver fat based on ultrasound techniques [54]. Conventional abdominal ultrasound examination lacks corresponding objective indicators, and the results of the diagnosis are affected by the patient’s body mass index (BMI), subcutaneous fat thickness, instrument sensitivity, and gain adjustment, resulting in the large discrepancies among different observers about MAFLD grading, especially in the evaluation of moderate and severe MAFLD [55, 56]. Current international guidelines do not recommend using ultrasound to stratify the severity of MAFLD [5]. Despite being considered as the gold standard in staging liver disease, the invasive nature of liver biopsy limits its use. To address this issue, non-invasive approaches have thus been developed. Even if computed tomography and magnetic resonance imaging can accurately detect and quantify liver fat, multiple limitations such as radiation, low availability, and high cost might affect the diagnostic feasibility [57, 58]. Therefore, increased non-invasive indexes of MAFLD have appeared. The included studies in this meta-analysis used the most widely applied complex score models, including the NAFLD fibrosis score (NFS) and fibrosis-4 (FIB-4) index to explore the link between the severity of liver fibrosis and colorectal tumors [59, 60]. However, advanced fibrosis are late manifestations. Detecting a progressive disease at an earlier stage would be beneficial. The indirect indexes of steatosis developed in recent years include the Fatty Liver Index [61], the Lipid Accumulation Product [62], the Hepatic Steatosis Index [63]. However, these indicators are not well suited for the diagnosis of steatosis grades [64]. Therefore, developing mature non-invasive scoring systems for liver fat quantification is necessary.

In this meta-analysis, a significant relationship between the severity of MAFLD and colorectal neoplasms was found in cross-sectional studies, but not in cohort studies. Due to the fact that only three cohort studies were included in this study, it was difficult to reflect the real relationship. Further evidence from prospective cohort studies are required to confirm whether the severity of MAFLD has a influence on the risk of colorectal tumors. Besides, studies performed in non-Asian regions showed a statistically significant pooled effect for colorectal adenomas, while those in Asian regions showed inconsistent findings. As for the association between the severity of MAFLD and advanced colorectal neoplasia, all relevant studies were performed in the Asian region. The result showed that severe MAFLD led to an increased ocurrence of advanced colorectal neoplasia compared to mild MAFLD. There is a need for more research in non-Asian population to clarify the role of MAFLD severity in the advanced colorectal neoplasia.

MAFLD patients of different severity levels lack formal guidelines or recommendations regarding routine colorectal neoplasm screening, despite the fact that the close relationship between them has been confirmed by many clinical studies. Besides, studies discovered that the severity of MAFLD is related to the poor prognosis of colorectal cancer. Severe MAFLD independently increased the risk of liver metastasis from CRC and colorectal CRC-specific mortality [65, 66]. There are reasons to believe that MAFLD patients, especially those with severe liver disease, could substantially benefit from more earlier or frequent colonoscopy. However, before implementation, the cost-effectiveness of regular colonoscopy screening still needs to be considered and validated. Further evaluation is also required to determine the right time for initiating such screening.

### Study strengths and limitations

This meta-analysis provide the most comprehensive and up-to-date assessment on the relationship between the severity of MAFLD and colorectal tumors. Wide regional coverage was involved, including Asia, Europe, and North America. A variety of statistical methods were combined to confirm the reliability of the outcomes. Based on the comprehensive search, it is unlikely that any published studies have been omitted, and neither funnel plots nor formal statistical tests indicate a publication bias. The study also has some limitations. First, some inherent limitations of cross-sectional studies led to the impossibility to accurately determine the incidence of future events. The lack of well-designed prospective studies resulted in the true causality between liver disease severity and colorectal tumors cannot be confirmed. Second, nearly half of the eligible studies did not fully adjust important confounding factors (such as obesity, metabolic syndrome, drug use, family history of cancer, etc.), so the risk of bias could not be ruled out, which could affect the reliability of the result. Third, significant heterogeneity among the eligible studies made it necessary to be cautious in interpreting some of the results of this meta-analysis. To systematically investigate and identify possible statistical heterogeneity sources, subgroup, meta-regression and sensitivity analyses were conducted. While meta-regression found that the heterogeneity was partly caused by the sex ratio, it was not possible to identify all possible heterogeneity due to the lack of detailed reports. The pooled subject data from large prospective studies is necessary for more thorough analysis of heterogeneity, as these become available over time. Fourth, MAFLD was diagnosed through liver biopsy in only five studies among the included studies. Liver biopsy provides the most accurate outcomes for diagnosing and staging MAFLD. However, invasive examinations are often not accepted by asymptomatic MAFLD patients. Furthermore, most of the included studies were from Asian countries. As the body fat distribution, genetic background, and living habits might significantly affect on the development of tumors in Asian and non-Asian individuals, the European and American populations should be studied in greater detail in prospective cohort studies.

## Conclusion

According to the findings of this study, MAFLD severity is independently related to colorectal adenomas and advanced colorectal neoplasia. Additionally, the left colon tumors are more likely to be caused by severe MAFLD, compared to the right colon tumors. Hence, patients with greater severity of MAFLD need a regular colonoscopy to detect colorectal tumors early and increase life expectancy. Perhaps regular colonoscopy screening in the future could help reduce the economic burden on society. A mechanism for this association needs to be investigated further.

## Supplementary Information

**Additional file 1.**

## Data Availability

All data generated or analyzed during this study are included in this published article.

## Abbreviations

AHRQ: Agency for Healthcare Research and Quality
aOR: Adjusted OR
BMI: Body mass index
CAP: Controlled Attenuation Parameter
CI: Confidence interval
CNKI: China National Knowledge Infrastructure
CRC: Colorectal cancer
FIB-4: Fibrosis-4
MAFLD: Metabolic dysfunction-associated fatty liver disease
NAFL: Non-alcoholic fatty liver
NAFLD: Non-alcoholic fatty liver disease
NASH: Nonalcoholic steatohepatitis
NFS: NAFLD fibrosis score
NOS: Newcastle-Ottawa Scale
OR: Odds ratio
TE: Transient Elastography
