# Supplementary material for: Association between the severity of metabolic dysfunction-associated fatty liver disease and the risk of colorectal neoplasm: a systematic review and meta-analysis
Source: Lipids Health Dis. 2022 Jun 6;21:52. doi: 10.1186/s12944-022-01659-1 (PMC9172084; doi:10.1186/s12944-022-01659-1)
Supplement: Supplementary file 1 — Additional file 1. [file 12944_2022_1659_MOESM1_ESM.docx]

# **Supplemental Table 1.** Search strategy of English database (from inception to 24^th^ April 2022)

| **Data source** | **Search terms** |
| --- | --- |
| PubMed | ((MAFLD) OR (Metabolic Dysfunction-associated Fatty Liver Disease) OR (("Non-alcoholic Fatty Liver Disease"[Mesh]) OR (((((((((((((Non alcoholic Fatty Liver Disease) OR (NAFLD)) OR (Nonalcoholic Fatty Liver Disease)) OR (Fatty Liver, Nonalcoholic)) OR (Fatty Livers, Nonalcoholic)) OR (Liver, Nonalcoholic Fatty)) OR (Livers, Nonalcoholic Fatty)) OR (Nonalcoholic Fatty Liver)) OR (Nonalcoholic Fatty Livers)) OR (Nonalcoholic Steatohepatitis)) OR (Nonalcoholic Steatohepatitides)) OR (Steatohepatitides, Nonalcoholic)) OR (Steatohepatitis, Nonalcoholic)))) AND ((("Adenomatous Polyposis Coli"[Mesh]) OR ((((((((((((((Adenomatous Polyposis Colus) OR (Coli, Adenomatous Polyposis)) OR (Colus, Adenomatous Polyposis)) OR (Polyposis Coli, Adenomatous)) OR (Polyposis Colus, Adenomatous)) OR (Adenomatous Polyposis of the Colon)) OR (Polyposis Coli)) OR (Coli, Polyposis)) OR (Colus, Polyposis)) OR (Polyposis Colus)) OR (Polyposis, Adenomatous Intestinal)) OR (Adenomatous Intestinal Polyposes)) OR (Adenomatous Intestinal Polyposis)) OR (Intestinal Polyposis, Adenomatous))) OR (("Colorectal Neoplasms"[Mesh]) OR (((((((((((((((Colorectal Neoplasm) OR (Neoplasm, Colorectal)) OR (Neoplasms, Colorectal)) OR (Colorectal Tumors)) OR (Colorectal Tumor)) OR (Tumor, Colorectal)) OR (Tumors, Colorectal)) OR (Colorectal Cancer)) OR (Cancer, Colorectal)) OR (Cancers, Colorectal)) OR (Colorectal Cancers)) OR (Colorectal Carcinoma)) OR (Carcinoma, Colorectal)) OR (Carcinomas, Colorectal)) OR (Colorectal Carcinomas)))) |
| EMBASE | ('metabolic fatty liver'/exp OR 'nonalcoholic fatty liver'/exp OR 'nonalcoholic fatty liver disease':ti,ab OR 'metabolic dysfunction-associated fatty liver disease':ti,ab OR 'metabolic fatty liver':ti,ab OR 'fatty liver, nonalcoholic':ti,ab OR 'fatty livers, nonalcoholic':ti,ab OR 'liver, nonalcoholic fatty':ti,ab OR 'livers, nonalcoholic fatty':ti,ab OR 'nonalcoholic fatty liver':ti,ab OR 'nonalcoholic fatty livers':ti,ab OR 'nonalcoholic steatohepatitis':ti,ab OR 'nonalcoholic steatohepatitides':ti,ab OR 'steatohepatitides, nonalcoholic':ti,ab OR 'steatohepatitis, nonalcoholic':ti,ab) AND ('colorectal tumor'/exp OR 'adenomatous polyposis colus':ti,ab OR 'coli, adenomatous polyposis':ti,ab OR 'colus, adenomatous polyposis':ti,ab OR 'polyposis coli, adenomatous':ti,ab OR 'polyposis colus, adenomatous':ti,ab OR 'adenomatous polyposis of the colon':ti,ab OR 'colon polyposis':ti,ab OR 'coli, polyposis':ti,ab OR 'colus, polyposis':ti,ab OR 'polyposis colus':ti,ab OR 'polyposis, adenomatous intestinal':ti,ab OR 'adenomatous intestinal polyposes':ti,ab OR 'adenomatous intestinal polyposis':ti,ab OR 'intestinal polyposis, adenomatous':ti,ab OR 'colorectal neoplasms':ti,ab OR 'colorectal neoplasm':ti,ab OR 'neoplasm, colorectal':ti,ab OR 'neoplasms, colorectal':ti,ab OR 'colorectal tumors':ti,ab OR 'tumor, colorectal':ti,ab OR 'tumors, colorectal':ti,ab OR 'colorectal cancer':ti,ab OR 'cancer, colorectal':ti,ab OR 'cancers, colorectal':ti,ab OR 'colorectal cancers':ti,ab OR 'colorectal carcinoma':ti,ab OR 'carcinoma, colorectal':ti,ab OR 'carcinomas, colorectal':ti,ab OR 'colorectal carcinomas':ti,ab) |
| Cochrane Library | #1 MeSH descriptor: [Non-alcoholic Fatty Liver Disease] explode all trees  #2 (MAFLD):ti,ab,kw OR (Non alcoholic Fatty Liver Disease):ti,ab,kw OR (NAFLD):ti,ab,kw OR (Nonalcoholic Fatty Liver Disease):ti,ab,kw OR (Fatty Liver, Nonalcoholic):ti,ab,kw (Word variations have been searched)  #3 (Fatty Livers, Nonalcoholic):ti,ab,kw OR (Liver, Nonalcoholic Fatty):ti,ab,kw OR (Livers, Nonalcoholic Fatty):ti,ab,kw OR (Nonalcoholic Fatty Liver):ti,ab,kw OR (Nonalcoholic Fatty Livers):ti,ab,kw (Word variations have been searched)  #4 (Nonalcoholic Steatohepatitis):ti,ab,kw OR (Nonalcoholic Steatohepatitides):ti,ab,kw OR (Steatohepatitides, Nonalcoholic):ti,ab,kw OR (Steatohepatitis, Nonalcoholic):ti,ab,kw (Word variations have been searched)  #5 (Metabolic Dysfunction-associated Fatty Liver Disease):ti,ab,kw  #6 #1 or #2 or #3 or #4 or #5  #7 MeSH descriptor: [Adenomatous Polyposis Coli] explode all trees  #8 (Adenomatous Polyposis Colus):ti,ab,kw OR (Coli, Adenomatous Polyposis):ti,ab,kw OR (Colus, Adenomatous Polyposis):ti,ab,kw OR (Polyposis Coli, Adenomatous):ti,ab,kw (Word variations have been searched)  #9 (Polyposis Colus, Adenomatous):ti,ab,kw OR (Adenomatous Polyposis of the Colon):ti,ab,kw OR (Polyposis Coli):ti,ab,kw OR (Coli, Polyposis):ti,ab,kw (Word variations have been searched)  #10 (Colus, Polyposis):ti,ab,kw OR (Polyposis Colus):ti,ab,kw OR (Polyposis, Adenomatous Intestinal):ti,ab,kw OR (Adenomatous Intestinal Polyposes):ti,ab,kw (Word variations have been searched)  #11 (Colus, PolyposisAdenomatous Intestinal Polyposis):ti,ab,kw OR (Intestinal Polyposis, Adenomatous):ti,ab,kw (Word variations have been searched)  #12 #7 or #8 or #9 or #10 or #11  #13 MeSH descriptor: [Colorectal Neoplasms] explode all trees  #14 (Colorectal Neoplasm):ti,ab,kw OR (Neoplasm, Colorectal):ti,ab,kw OR (Neoplasms, Colorectal):ti,ab,kw OR (Colorectal Tumors):ti,ab,kw OR (Colorectal Tumor):ti,ab,kw (Word variations have been searched)  #15 (Tumor, Colorectal):ti,ab,kw OR (Tumors, Colorectal):ti,ab,kw OR (Colorectal Cancer):ti,ab,kw OR (Cancer, Colorectal):ti,ab,kw OR (Cancers, Colorectal):ti,ab,kw (Word variations have been searched)  #16 (Colorectal Cancers):ti,ab,kw OR (Colorectal Carcinoma):ti,ab,kw OR (Carcinoma, Colorectal):ti,ab,kw OR (Carcinomas, Colorectal):ti,ab,kw OR (Colorectal Carcinomas):ti,ab,kw (Word variations have been searched)  #17 #12 or #13 or #14 or #15 or #16  #18 #12 or #17  #19 #6 and #18 |
| Web of Science | #1 TS=(MAFLD OR Metabolic Dysfunction-associated Fatty Liver Disease OR Non-alcoholic Fatty Liver Disease OR Non alcoholic Fatty Liver Disease OR NAFLD OR Nonalcoholic Fatty Liver Disease OR Fatty Liver, Nonalcoholic OR Fatty Livers, Nonalcoholic OR Liver, Nonalcoholic Fatty OR Livers, Nonalcoholic Fatty OR Nonalcoholic Fatty Liver OR Nonalcoholic Fatty Livers OR Nonalcoholic Steatohepatitis OR Nonalcoholic Steatohepatitides OR Steatohepatitides, Nonalcoholic OR Steatohepatitis, Nonalcoholic)  #2 TS=(Adenomatous Polyposis Coli OR Adenomatous Polyposis colds OR Coli, Adenomatous Polyposis OR colds, Adenomatous Polyposis OR Polyposis Coli, Adenomatous OR Polyposis colds, Adenomatous OR Adenomatous Polyposis of the Colon OR Polyposis Coli OR Coli, Polyposis OR colds, Polyposis OR Polyposis colds OR Polyposis, Adenomatous Intestinal OR Adenomatous Intestinal polyposis OR Adenomatous Intestinal Polyposis OR Intestinal Polyposis, Adenomatous OR Colorectal Neoplasms OR Colorectal Neoplasm OR Neoplasm, Colorectal OR Neoplasms, Colorectal OR Colorectal Tumors OR Colorectal Tumor OR Tumor, Colorectal OR Tumors, Colorectal OR Colorectal Cancer OR Cancer, Colorectal OR Cancers, Colorectal OR Colorectal Cancers OR Colorectal Carcinoma OR Carcinoma, Colorectal OR Carcinomas, Colorectal OR Colorectal Carcinomas)  #1 AND #2 |
